# Computational Exploration of the Effects of Mutations on GABA Aminotransferase in GABA Aminotransferase Deficiency

**DOI:** 10.3390/ijms241310933

**Published:** 2023-06-30

**Authors:** Muhammad Yasir, Jinyoung Park, Eun-Taek Han, Won Sun Park, Jin-Hee Han, Yong-Soo Kwon, Hee-Jae Lee, Wanjoo Chun

**Affiliations:** 1 Department of Pharmacology, Kangwon National University School of Medicine, Chuncheon 24341, Republic of Korea; yasir.khokhar1999@gmail.com (M.Y.); jinyoung0326@kangwon.ac.kr (J.P.); heejaelee@kangwon.ac.kr (H.-J.L.); 2 Department of Medical Environmental Biology and Tropical Medicine, Kangwon National University School of Medicine, Chuncheon 24341, Republic of Korea; ethan@kangwon.ac.kr (E.-T.H.); han.han@kangwon.ac.kr (J.-H.H.); 3 Department of Physiology, Kangwon National University School of Medicine, Chuncheon 24341, Republic of Korea; parkws@kangwon.ac.kr; 4 College of Pharmacy, Kangwon National University School of Medicine, Chuncheon 24341, Republic of Korea; yskwon@kangwon.ac.kr

**Keywords:** GABA-AT, molecular dynamic simulation, gmxMMPBSA free energy calculation, GABA-AT mutation

## Abstract

Gamma-aminobutyric acid (GABA) transaminase—also called GABA aminotransferase (GABA-AT)—deficiency is a rare autosomal recessive disorder characterized by a severe neonatal-infantile epileptic encephalopathy with symptoms such as seizures, hypotonia, hyperreflexia, developmental delay, and growth acceleration. GABA transaminase deficiency is caused by mutations in GABA-AT, the enzyme responsible for the catabolism of GABA. Mutations in multiple locations on GABA-AT have been reported and their locations have been shown to influence the onset of the disease and the severity of symptoms. We examined how GABA-AT mutations influence the structural stability of the enzyme and GABA-binding affinity using computational methodologies such as molecular dynamics simulation and binding free energy calculation to understand the underlying mechanism through which GABA-AT mutations cause GABA-AT deficiency. GABA-AT 3D model depiction was carried out together with seven individual mutated models of GABA-AT. The structural stability of all the predicted models was analyzed using several tools and web servers. All models were evaluated based on their phytochemical values. Additionally, 100 ns MD simulation was carried out and the mutated models were evaluated using RMSD, RMSF, R_g_, and SASA. gmxMMPBSA free energy calculation was carried out. Moreover, RMSD and free energy calculations were also compared with those obtained using online web servers. Our study demonstrates that P152S, Q296H, and R92Q play a more critical role in the structural instability of GABA-AT compared with the other mutated models: G465R, L211F, L478P, and R220K.

## 1. Introduction

Gamma-aminobutyric acid (GABA) transaminase deficiency is a rare disease of GABA metabolism associated with aberrant development, seizures, and an inflated level of GABA in the brain. Children with a GABA-transaminase deficiency have significant developmental impairments [1,2]. GABA is an inhibitory neurotransmitter that is abundant in the brain and is produced from glutamate in presynaptic GABAergic neurons. Depolarization of presynaptic neurons stimulates GABA release for neurotransmission [3]. The two main GABA receptors (GABA_A_ and GABA_B_) on the postsynaptic neuron can bind to GABA once it has been released into the synapses. As a result of allosteric GABA binding to GABA_A_ receptors, the central chloride ion channel of the receptor opens, causing the neuronal membrane to become hyperpolarized. This produces a reduction in cell excitability and, as a result, neural inhibition [4,5]. GABA_B_ receptors are metabotropic; upon activation by GABA, associated potassium channels are opened by G-protein coupled receptors, causing hyperpolarization and neuronal inhibition similar to that of GABA_A_ receptors [6]. GABA’s function is halted after synaptic neurotransmission either by reuptake into presynaptic neurons or by absorption into glial cells through GABA transporters [7]. In glial cells, GABA is catabolized by GABA-AT (also called GABA-transaminase (GABA-T)), 4-aminobutyrate transaminase, and 4-aminobutyrate aminotransferase [8,9].

GABA-AT is a member of a big family of pyridoxal 5′-phosphate (PLP)-dependent aminotransferases and catalyzes the breakdown of GABA into succinic semialdehyde [10]. The Lys329-bound (Lys357 in human) PLP coenzyme undergoes a transformation to pyridoxamine 5′-phosphate (PMP) during this enzymatic process; as a result, the enzyme does not serve as a catalyst when PLP is converted to PMP since the enzyme is altered. To attain catalyst status, a second catalytic phase is necessary for which the enzyme employs a second substrate, α-ketoglutarate, to convert the PMP to PLP so that catalysis can restart [11]. As a result of that process, the excitatory neurotransmitter glutamate is generated from the α-ketoglutarate [12]. Therefore, during the whole process, one GABA molecule is converted into one glutamate molecule. The mechanism for converting PMP back to PLP is the converse of the mechanism that converts PLP to PMP. Since glutamate is converted to GABA by the PLP-dependent enzyme glutamic acid decarboxylase (GAD), the two PLP-dependent enzymes GABA-AT and GAD are crucial for controlling the levels of these two neurotransmitters in the brain [13]. Under normal conditions, convulsions can be triggered when the level of GABA diminishes below a threshold level in the brain and seizures can be stopped by boosting GABA levels. Therefore, inhibiting GABA-AT with inhibitors such as vigabatrin has been shown to successfully reduce excessive neural activity in patients with epilepsy [14,15]. However, in GABA aminotransferase deficiency disorders, where endogenous GABA levels are aberrantly elevated due to defective mutations in GABA-AT, the occurrence of seizures increases, resulting in a condition known as epileptic encephalopathy [16]. The seizure-inducing effects of persistently elevated GABA levels in patients with GABA-AT deficiency are still not understood. Putative hypotheses have been proposed, including excessive inhibition of inhibitory interneurons, paradoxical depolarizing effects, and downregulation of GABA receptors, as demonstrated in succinic semialdehyde dehydrogenase deficiency, another disorder affecting GABA metabolism [17]. GABA-AT deficiency is caused by recessive mutations in the ABAT gene (MIN 137150) [16]. Since the first report of GABA-AT deficiency in 1984, more cases have been reported. Mutations reported in patients have been identified in several locations, including R92Q, P152S, L211F, R220K, Q296H, G465R, and L478P. There are instances where patients have more than one mutation. The locations of mutations have been shown to influence the onset, type, and severity of seizures in patients. Mutations like R220K and Q296H lead to the onset of the condition at birth, while the emergence of P152S and R92Q mutations is observed at 6 months and 7 months old, respectively [18].

In the present study, we examined the effects of GABA-AT mutations on the structural stability of the enzyme and the affinity of binding to GABA using computational tools such as molecular dynamics simulation and gmxMMPBSA analyses to understand the underlying mechanism through which GABA-AT mutants cause GABA-AT deficiency disorders.

## 2. Results and Discussion

### 2.1. Mechanism of Action of GABA-AT

**Figure 1:**
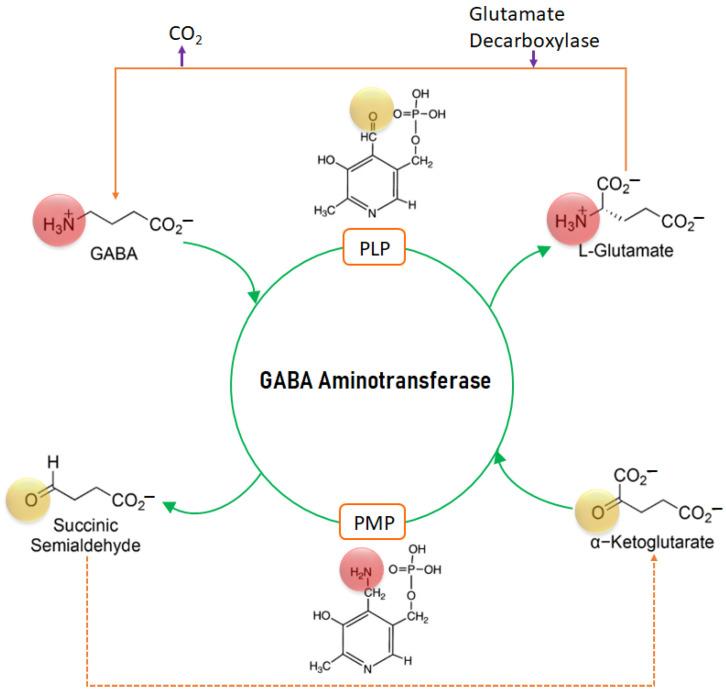
The mechanism of action of GABA-AT.

The mechanism of action of GABA-AT is depicted in Figure 1. PLP (pyridoxal 5′-phosphate) binds to GABA and converts it to succinic semialdehyde (SSA) and is itself converted to PMP (pyridoxamine 5′-phosphate) by taking the amino group. PMP then binds to α-ketoglutarate and converts it to L-Glutamate by donating the amino group, in the process converting to PLP. The glutamate decarboxylase enzyme further converts L-Glutamate to GABA by releasing CO_2_.

### 2.2. Prediction and Evaluation of Mutated Tyrosinase Structures

Human GABA-AT is a homodimeric protein consisting of 461 amino acid residues. The Human GABA amino acid sequence was retrieved from the UniProt database (UniPort ID P80404). The 3D structure of human GABA-AT was predicted using SWISS-MODEL, using the structure of *Sus scrofa* (PDB ID 4Y0H) as the reference (Supplementary Data Figure S1). The reference structure of *Sus scrofa* showed 95.67% sequence identity with human GABA-AT and 1.63 Å X-ray resolution. Furthermore, seven different mutant structures were predicted by substituting certain residues at specific positions within the wild amino-acid sequences of human GABA. A total of two Arginine (R) residues at positions 92 and 220 were mutated into Glutamine (Q) and Lysine (K), respectively, and two Leucine residues were mutated in Phenylalanine (F) and Proline (P) at locations 211 and 478. Furthermore, Glycine (G), Proline (P), and Glutamine (Q) were mutated into Arginine (R), Serine (S), and Histidine (H) at positions 465, 152, and 296, respectively.

**Figure 2:**
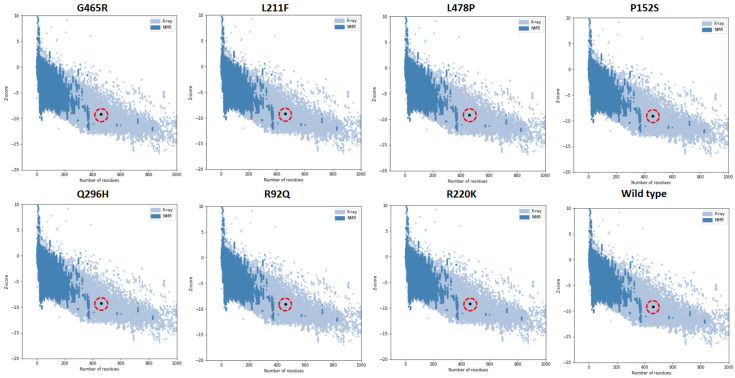
Graphical depiction of ProSA results comparing the mutated models with X-ray and NMR structures available in PDB. The location of our modeled structure is highlighted using the red circle.

Several online servers and tools, including MolProbity, ProSA, and ERRAT were used for structural optimization and assessment in order to gauge the precision and dependability of the anticipated models. MolProbity scores take into account multiple factors such as bond lengths, bond angles, dihedral angles, clash scores, and other geometric parameters. The model score was used to evaluate the predictability and efficacy of the anticipated models. The MoProbity predicted score values of 68, 85, 87, 89, 88, 89, 89, and 89% for G465R, L211F, L478P, P152S, Q296H, R92Q, R220K, and the wild type (Table 1). Ramachandran analysis showed that most of the amino acid residues were present in favorable and allowed regions, with all the mutated models showing more than 90% amino acid residues in the favored region. The graphical presentation of Ramachandran plots of all predicted mutated structures are shown in Supplementary Data Figure S2. The overall quality score of the predicted models was also analyzed using ERRAT scores and ProSA. The z-score values indicate the overall model quality of the anticipated mutated structures of G465R, L211F, L478P, P152S, Q296H, R92Q, R220K, and the wild type as −9.14, −9.17, −9.16, −9.05, −9.28, −9.12, −9.21, and −9.27, respectively. Moreover, VADAR statistical analysis of the protein revealed that the human GABA-AT protein contained 39% α-helix, 19% β-sheets, 41% coil, and 19% turns. The graphical representations of ProSA graphs of all anticipated models are shown in (Figure 2).

### 2.3. Residual Mutation Analysis

**Figure 3:**
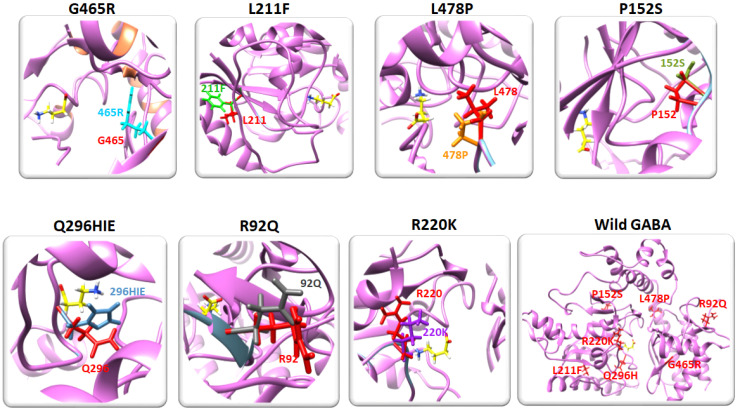
The superimposed mutated models are shown in this figure. The wild amino acid residues are colored red while the mutated residues are indicated in different colors. The locations of all mutated residues in wild type GABA-AT are also shown.

The predicted mutated models were analyzed using Discovery Studio and UCSF Chimera to evaluate the impact of mutated amino acid residues on the 3D conformation and folding of the protein. Superimposition of the wild and mutated models showed that the R220K and Q296H mutations were located close to the binding pocket of GABA-AT, with a minimal distance of 10.35 and 6.49, respectively, and may affect the binding of the ligand (GABA) to the modeled protein (GABA-AT). The graphical depictions of all the mutated models are shown in Figure 3.

### 2.4. Prediction of the Physiochemical Properties of the Predicted Mutated Models

Prediction of the physiochemical properties of the projected mutant models were carried out using ProtParam. The theoretical pl was computed using pK values of the amino acids. The results revealed that L211F, L478P, P152S, Q296H, and the wild type had the same pI value (7.31) while G465R, R92Q, and R220K had pI values of 7.68, 6.98, and 7.30, respectively, which were comparable to the benchmark values, demonstrating the precision of the anticipated structures. Additionally, the anticipated aliphatic index value showed the volume and stability of aliphatic side chains. The estimated GRAVY value represented the total of all residues’ hydropathy values; more hydrophilic and less hydrophobic behaviors of protein structures are shown by negative GRAVY values (Table 2). In contrast to human wild type GABA, the projected physiochemical attributes showed the dependability, effectiveness, and stability of the anticipated model structure of mutant GABA. The reasonableness of all the projected values showed the precision of the predicted structures.

### 2.5. Prediction of GABA-AT Binding Site

**Figure 4:**
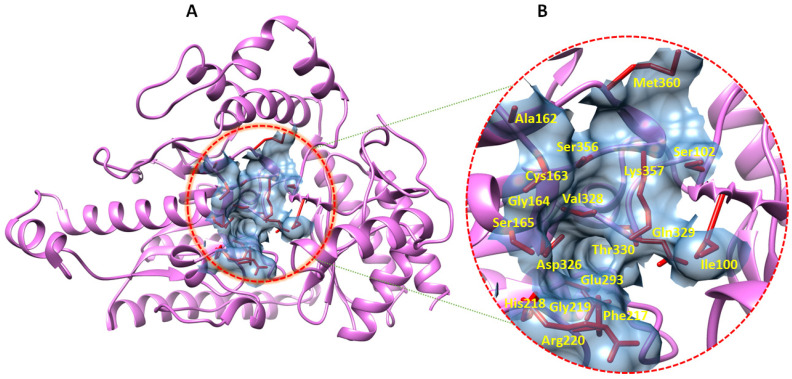
(**A**,**B**). The graphical representation of human wild type GABA-AT shows the location of the binding pocket (**A**). The predicted amino acid residues are shown and labeled in yellow in their respective location (**B**).

The function of a binding pocket is dictated by the assemblage of amino acid residues around it, as well as its shape and placement inside a protein [19,20]. P2Rank, an exciting technique for predicting ligand binding sites, is accessible through PrankWeb, a website resource. Therefore, from 11 predicted binding pockets, the topmost binding pocket was selected based on the highest scoring value (Table 3). The scoring values are based on the probability of binding pocket scores. The full-length table is provided in Supplementary Data Table S1. Therefore, the selected binding pocket residues were Ile100, Ser102, Ala162, Cys163, Gly164, Ser165, Phe217, His218, Gly219, Arg220, Glu293, Asp326, Val328, Gln329, Gln330, Ser356, Lys357, and Met360 (Figure 4A,B). Furthermore, the predicted binding pocket residues were compared to *Sus scrofa* binding pocket amino acid residues by superimposing both proteins for structural and binding pocket validation (Supplementary Data Figure S3).

### 2.6. Molecular Dynamics Simulations

The predicted models of all seven mutations were subjected to 100 ns MD simulations to analyze the residual flexibility and stability of mutated structures.

#### 2.6.1. Root Mean Square Deviation Analysis

**Figure 5:**
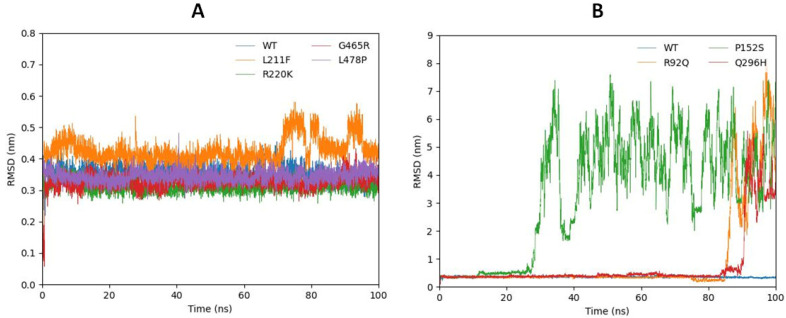
(**A**,**B**). Stable RMSD values of L211F (orange), R220K (green), G465R (red), and L478P (purple) compared with the wild type (blue) are represented in (**A**) while the highly fluctuating mutations R92Q (orange), P152S (green), and Q296H (red) compared with the wild type (blue) are predicted in (**B**).

To analyze the structural behavior of ligands in the active region of mutated proteins in comparison to the wild type, RMSD analysis was carried out from 100 ns MD trajectories. RMSD analysis revealed that mutated residues P (Proline), R (Arginine), and Q (Glutamine) change the structural behavior of GABA by exhibiting a huge number of fluctuations in ligand interactions. The predicted wild type, R220K, G465R, and L478P structures showed the most stable behavior and maintained the RMS deviation point at σ = 0.35 in RMSD analysis, while L211F showed increasing behavior at the start; however, after reaching 10 ns, the bar line showed a decrease in values and remained stable until 70 ns. After reaching 70 ns, the bar line increased the RMSD values and kept fluctuating until 90 ns and maintained its initial position at RMSD σ = 0.4 from 90 ns to 100 ns. On the other hand, the most fluctuating mutated models are depicted in a separate graph in comparison to the wild model (Figure 5A,B). Mutated model P152S indicated the most unstable behavior as it remained steady until 12 ns and then started to increase RMSD values. After 28 ns, the bar line fluctuated drastically and kept fluctuating until 100 ns. Furthermore, R92Q and Q296H both exhibited stable bar lines until 82 ns and then the bar line fluctuated, increasing the RMSD values. Moreover, triplicate runs were conducted to show the statistical validity of the predicted results and the graphs (Supplementary Data Figure S4) show behavior that is quite similar to that in Figure 5. The stable RMSD values of R220K, G465R, L478P, and L211F are due to the stability of the backbone of the mutated protein, while the higher RMSD values of Q296H, R92Q, and P152S are due to the fluctuating or unstable backbone of the modeled protein.

#### 2.6.2. Root Mean Square Fluctuations

**Figure 6:**
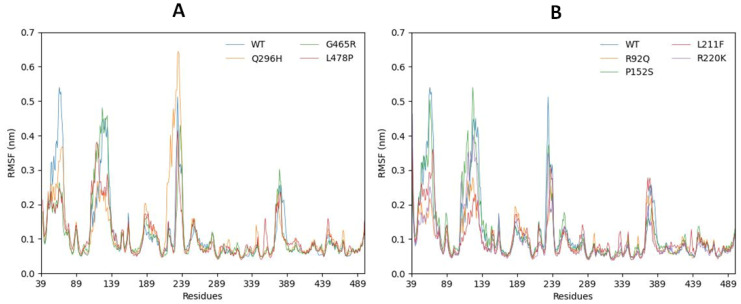
(**A**,**B**). RMSF graphs of anticipated mutated structures from res39 to res489.

**Figure 7:**
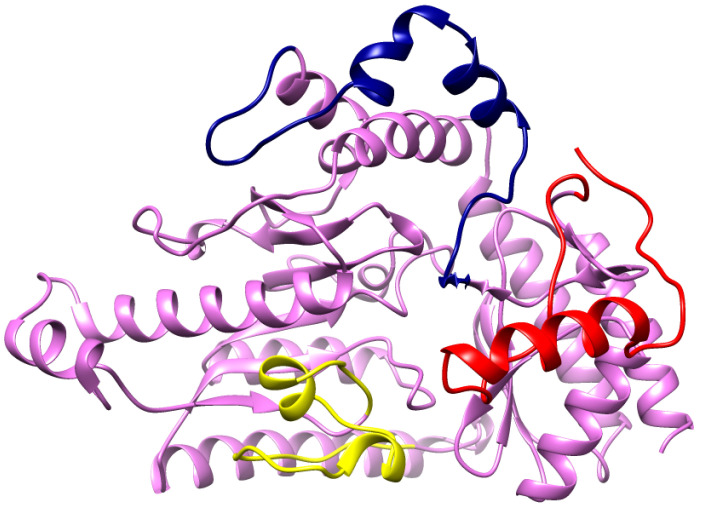
Highly fluctuating regions in RMSF are shown in the 3D model. All the fluctuating regions are indicated in different colors. Residues 47–80 are colored red, residues 109–140 are colored blue, and residues 230–241 are colored yellow.

The RMSF results of all the mutant models fluctuated dynamically from residue 39 to the C terminals. A total of three peaks were observed in the graphical representation of the RMSF, with two peaks of the mutated models Q296H and P152S being higher than 0.55 nm. The Q296H mutant model showed the highest fluctuating peak, reaching 0.65 nm; the peak for P152S reached 0.55 nm. Therefore, all depicted models showed fluctuation ranges of 47–80, 109–140, and 230–241 for the residues. Furthermore, a small peak was predicted at 378–389, as shown in Figure 6A,B. The comparative results showed that Q296H and P152S contributed more to RMS fluctuation compared with the other predicted models, with a higher impact on the structural behavior of the 3D protein (Figure 6A,B). Furthermore, amino acid residues with high RMS peaks in the wild type protein were assessed and analyzed in a 3D model. The 3D model shows that most loop regions show higher fluctuating peaks (Figure 7). These findings prove that mutations at these positions (296, 152) modulate the structural confirmation of the predicted mutated models, which may cause instability, disturbance, and the respective downstream signaling pathway.

#### 2.6.3. Radius of Gyration (R_g_)

**Figure 8:**
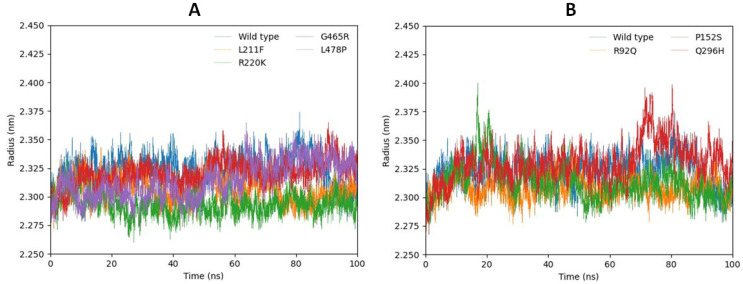
(**A**,**B**). Stable R_g_ values of L211F, R220K, G465R, and L478P compared with the wild type are shown in (**A**) while fluctuating R_g_ values of P152S and Q296H compared with R92Q and the wild type are shown in (**B**).

The structural compactness of a protein structure can be calculated using the radius of gyration (R_g_). Stably folded proteins show relatively steady values over the 100 ns simulation time while misfolded or unstable proteins show fluctuations during the simulation time span. The resulting value indicates that the Rg values of the mutated models L211F, R220K, G465R, L478P, and the wild type have similar types of fluctuation patterns and remain stable between 2.87 nm and 2.350 nm during the 100 ns MD simulation. Furthermore, the mutated models Q296H and P152S predicted highly fluctuating peaks from 70 ns to 90 ns and at 18 ns, respectively, and relatively different fluctuating behavior compared with R92Q and the wild type. The graphical representation shows that the residual backbone and 3D folding of the receptor protein were unstable in mutated models Q296H and P152S (Figure 8A,B).

#### 2.6.4. Solvent-Accessible Surface Area

**Figure 9:**
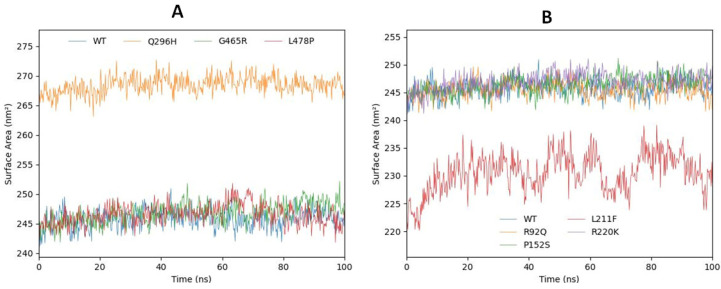
(**A**,**B**). The SASA values for Q296H, G465R, and L478P compared with the wild type are shown in (**A**). The SASA values for R92Q, L211F, R220K, and P152S compared with the wild type are shown in graph (**B**).

The solvent-accessible surface area (SASA) was also examined. The graphical depictions of the SASAs are shown in Figure 9A,B. The results revealed that the SASA values of all the mutated models are centered at 245 nm^2^ except Q296H and L211F. Q296H showed higher SASA values compared with all other mutated models and predicted wild type GABA model. On the contrary, L211F showed the lowest SASA values compared with R220K, G465R, L478P, Q296H, P152S, R92Q, and the wild type model. The higher SASA value of Q296H indicated the instability of the predicted model and showed that the large area of the mutated model was accessible to the solvent during 100 ns MD simulations, while the low SASA values of L211F showed that less surface area was accessible to the solvent during the 100 ns. R220K, G465R, L478P, P152S, R92Q, and the wild type models were predicted to have a similar behavior and the same slightly increasing pattern until 100 ns.

### 2.7. gmxMMPBSA Free Energy Calculation

**Figure 10:**
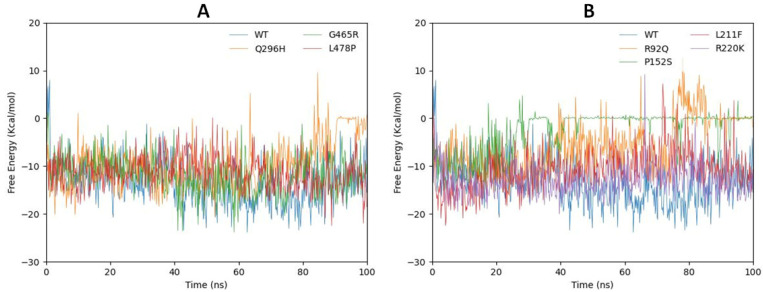
(**A**,**B**). The gmxMMPBSA free energy calculation for Q296H (orange), G465R (green), and L478P (red) compared with the wild type (blue) are depicted in (**A**). The graphical presentation of the free energy calculation of mutated models P92Q (orange), L211F (red), R220K (purple), and P152S compared with the wild type (blue) are depicted in (**B**).

A hundred nanosecond trajectories and topologies of complexes were extracted to calculate the binding free energy of the GABA complexes using the MMPBSA Approach. The mutated models Q296, G465R, and L478P compared with the wild type are shown in Figure 10A while mutated models R92Q, P152S, L211F, and R220K compared with the wild type are depicted in Figure 10B. The graphical presentation revealed that G465R and L46R remained stable compared with the wild type until the gmxMMPBSA free energy was calculated at 100 ns; however, Q296H fluctuated, with the bar line changing its position at 90 ns and showing high gmxMMPBSA free energy. Furthermore, in Figure 10B, L211F and R220K were stable compared with the wild type and had −10 gmxMMPBSA free energy, while R92Q and P152S showed different bar lines compared with the others. R92Q remained stable at the start; however, after reaching 40 ns, the bar line started fluctuating and the free energy increased. After 70 ns, the bar line increased dramatically, reaching a peak, Furthermore, positive energy values were observed, equilibrating at ΔG = 0 from 85 ns to 100 ns. The free energy values of the P152S mutated model started increasing from 12 ns, and at 30 ns, the bar line stabilized at ΔG = 0, remaining here until 100 ns.

### 2.8. Comparative Analysis

Structural differences due to the mutations were analyzed based on the measure of consensus RMSD values using the PDBeFold webserver. Mutations resulted in higher consensus RMSD values, which means that mutations cause structural dissimilarity of mutated proteins compared with the wild type protein (Table 4). However, the mutations did not cause a high disturbance in the contents and locations of the secondary structures such as α-helix and β-sheet. Furthermore, changes in Gibbs free energy due to the mutations were analyzed and compared with the wild type using the Elaspic webserver. All the mutations resulted in increased Gibbs free energy values, indicating that the mutations caused destabilization of the protein (Table 4), indicating that mutations result in a decrease in the stability of the protein. Furthermore, the increased free energy can also have a substantial impact on the affinity of binding between the protein and the ligand, GABA.

The Elaspic web server gave 2.94 ΔG values, which were not correlated with gmxMMPBSA free energy calculations. gmxMMPBSA employs a molecular dynamics simulation approach to calculate free energy differences. It involves running molecular dynamics simulations of a protein–ligand complex, solvating the system, and calculating the binding free energy using a combination of molecular mechanics force fields and solvation models. Elaspic, on the other hand, uses an elastic network model to predict changes in protein stability caused by mutations. It utilizes the protein’s structure and sequence information to estimate the impact of amino acid substitutions on the protein’s stability, folding, and interactions. Due to the different algorithms, variations may occur. Therefore, in the RMSD analysis, Q296H exhibited highly fluctuating RMSD values, and R92Q and L478P also exhibited high RMSD values following Q296H. In MD simulation, L478P demonstrated quite stable behavior in all RMSD, RMSF, Rg, SASA, and gmxMMPBSA, while R92Q and Q296H were found to fluctuate highly.

### 2.9. Structural Analysis at 100 ns of MD Simulation

**Figure 11:**
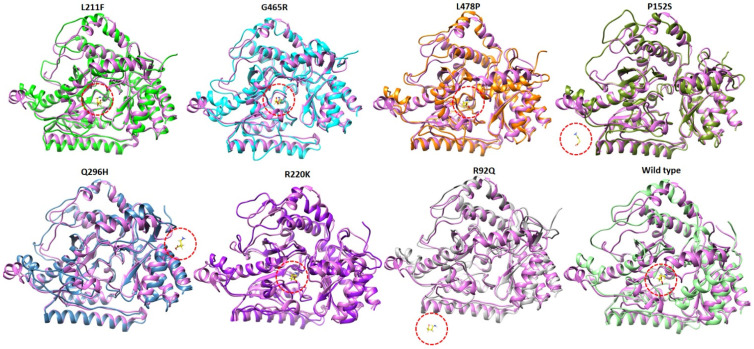
Superimposition of mutated models obtained at 100 ns on the wild type GABA-AT model. The wild protein is colored orchid while the other proteins are indicated in different colors. Furthermore, the ligand is colored yellow and the binding and removal of the ligand from the active region is highlighted with red circles.

The snapshots of all mutated models and their complexes with GABA were retrieved at 100 ns MD simulation to analyze the 3D structure and folding conformation of mutated models in comparison with the wild type. The mutated models obtained at 100 ns were superimposed on the wild type model’s protein to pinpoint the structural changes. The results showed that the mutated models P152S, R92Q, and Q296H showed structural instability and many parts of the proteins were unfolded. Furthermore, the ligand bound in the active region of the target protein was removed from the binding pocket (Figure 11). L211F, G465R, L478P, R220K, and the wild type showed almost the same structural pattern. The removal of the ligand from the active region of the target protein could be due to the loose folding and high fluctuation of the 3D structures of GABA-AT protein during 100 ns MD simulation.

## 3. Methods and Materials

### 3.1. Prediction of Human Tyrosinase Structure

GABA-AT is a homodimeric enzyme with 461 amino acid residues and a molecular weight of 56 kDa per monomer. The crystal structure of human GABA was not available on the Protein Data Bank (PDB) (https://www.rcsb.org/) (accessed on 1 March 2023). The primary structure of GABA-AT was deduced from the cDNA of the *Sus scrofa* brain. The *Sus scrofa* GABA-AT enzyme sequence has 95.67% homology with the *Homo sapiens* enzyme, which authenticates the studies with the *Sus scrofa* enzyme as being highly pertinent to humans.

Therefore, a homology modeling approach was used to predict the human GABA-AT 3D (three-dimensional) model. The *Sus scrofa’s* X-ray structure with 1.63 Å was designated as a template for the 3D structure prediction of human GABA-AT based on sequence similarity. The 3D structure of wild type human GABA was predicted using the online server, SWISS-MODEL (https://swissmodel.expasy.org/) (accessed on 3 March 2023). All the selected mutated (G465R, L211F, L478P, P152S, Q296H, R92Q, and R220K) structures were generated by modifying residual sites in the wild type structure using the PyMOL visualization tool. The constructed target proteins were minimized by Discovery Studio. Furthermore, the stereo-chemical characteristics of the anticipated mutant structures were evaluated using ProSA-web and the MolProbity server. Additionally, an online web server, ProtParam, was employed to predict the theoretical pI values, Aliphatic index (AI), and GRAVY values. Moreover, Ramachandran plots and values were predicted using the MolProbity server (http://molprobity.biochem.duke.edu/) (accessed on 15 March 2023). The overall protein architecture and the statistical percentage values of α-helices, β-sheets, coils, and turns were obtained from VADAR (http://vadar.wishartlab.com/) (accessed on 20 March 2023), an online server [21].

### 3.2. Residual Mutation Analysis

The chosen mutants were used to forecast how they might affect protein structures and the diseases they are connected with. The seven mutations G465R, L211F, L478P, P152S, Q296H, R92Q, and R220K were selected from the literature [18]. The mutated models were analyzed individually using Discovery Studio [22] and the UCSF Chimera tool [23].

### 3.3. Binding Pocket Prediction Analysis

The position of ligands in the holo-structure of a protein is known to determine the binding pocket of the desired protein [24]. PrankWeb (https://prankweb.cz/) (accessed on 25 March 2023), an online server, was employed to explore the probability of amino acid residues involved in the formation of active binding sites [25]. Furthermore, all the binding pockets predicted by PrankWeb were evaluated based on scoring values and ranked based on the confirmation of the pocket in the holo-structure of the protein.

### 3.4. Molecular Dynamic Simulation

All seven mutated proteins and the modeled wild type GABA protein were subjected to 100 ns MD simulation to analyze the residual flexibility of the predicted mutated structure in comparison with the wild type GABA.

The CHARMM-GUI server’s solution builder tool was used to generate the CHARMM36 force field, and the same procedure was also used to create the input files for MD simulations in GROMACS. As a result, five steps were accomplished: in the 1st step, the GABA-AT in complex with GABA was uploaded to the CHARMM-GUI server; in the 2nd step, TIP3P solution was used to solvate the existing model into a periodic, rectangular box that was extended 10 beyond each peptide’s atom. The basic ion type chosen was KCL, the ion concentration was set by default to 0.15, and the Monte-Carlo ion implementing method was used. The counter ions were added up until the system was neutralized. The box’s dimensions were adjusted in the 3rd step (solvator) to 94 along each axis (A, B, and C), giving a total system size of around 830,584 cubic Angstroms. The LINCS method was used to restrict bonds and the Verlet cutoff approach with 10 Å was used for electrostatic and Van der Waals interactions. Additionally, the electrostatic interactions were calculated using the particle mesh Ewald (PME) approach. The steepest descent method for energy minimization was used for the solvated systems. In the 4th step, systems underwent two phases of equilibration. The systems were subjected to the NVT condition before being brought to equilibrium in the NPT condition, and the simulation temperature was adjusted at 30 °C. In order to produce GROMACS topology (top) and parameter (itp) files, the CHARMM-GUI offers a Python format conversion tool at the 5th step for MD simulations in GROMACS. The structural behavior of protein and ligand complexes was investigated using the Linux operating system and the GROMACS tool (version 2019.3). GROMACS was used to run the production dynamics with a 2 fs time step, and the coordinates for each picosecond were stored in a file for MD analysis.

### 3.5. Free Energy Calculation Using gmxMMPBSA

One of the most often used techniques for calculating binding free energy is molecular mechanics/Poisson-Boltzmann (Generalized-Born) surface area. It has been demonstrated that this approach balances precision with computational effectiveness, particularly when working with big systems [26]. Therefore, Free energy calculation was carried out using the gmxMMPBSA tool, utilizing Gromacs trajectory and topology files. An index file was generated at the start and the output was saved in DAT (.dat) and CSV (.csv) format. The results were analyzed using the gmxMMBSA ana module.

### 3.6. Comparative Analysis of Mutated Structures

The role of mutations in protein structure and stability was further examined using PDBeFold and Eplaspic web servers. PDBeFold is a web server for predicting the structure of proteins from their sequences, developed by the Protein Data Bank in Europe (PDBe) (https://www.ebi.ac.uk/msd-srv/ssm/) (accessed on 4 April 2023). PDBeFold uses a variety of methods to predict protein structures, including homology modeling, threading, and ab initio structure prediction [27]. PDBeFold can be used to identify the role of mutations in protein structures by measuring consensus RMSD values. Consensus RMSD, the average of the RMSD values obtained from mutant sequences and structure superpositions, indicates the degree of structural differences. The greater the difference in consensus RMSD, the greater the difference in the protein structures. Wild type and seven mutants of GABA aminotransferase were uploaded to the PDBeFold web server, and the structural differences were analyzed using the consensus RMSD values. Moreover, the ELASPIC webserver (http://elaspic.kimlab.org/) (accessed on 5 April 2023), a computational tool, was employed for predicting the effects of mutations on protein stability and protein–protein interactions [28] The ELASPIC webserver uses a combination of machine learning and molecular modeling techniques to predict the effect of mutations on the stability and interactions of a protein. The difference in free energy (ΔG) is used to predict the effect of a mutation on protein stability. A positive ΔG indicates that the mutant is less stable than the wild type and the magnitude of the ΔG is used to predict the severity of the effect on protein stability.

## 4. Conclusions

In GABA aminotransferase deficiency disorders, where the endogenous GABA level is aberrantly elevated due to the defective mutations in GABA-AT, the occurrence of seizures increased, resulting in a condition known as epileptic encephalopathy. In this study, we analyzed the structural and 3D folding behavior of wild type human GABA-AT and seven mutations associated with GABA-AT. Our study reveals that, of the seven individual mutations (G465R, L211F, L478P, P152, Q296H, R92Q, and R220K), only P152S, Q296H, and P152S cause more structural instability of the protein in RMSD, RMSF, R_g_, and gmxMMPBSA free energy calculations. However, no bigger structural changes were observed in the β-sheets of the protein, although loop structures are mostly affected due to the mutations. R220K is the mutation closest to the binding region of GABA-AT and does not cause any hindrance to the binding of GABA (ligand) to the respective protein (GABA-AT). On the other hand, Q296H, R92Q, and P152S cause detachment of the ligand from the active region of the target protein.

## Figures and Tables

**Table 1:** Structural validation values of seven mutated models compared with the wild type GABA-AT.

Sr No	Mutated Structures	MolProbity Score	ProSA Z-Score	ERRAT Score	Ramachandran Favored
1	G465R	2.13	−9.14	89.95	90.41%
2	L211F	1.80	−9.17	87.26	90.63%
3	L478P	1.75	−9.16	91.27	90.63%
4	P152S	1.70	−9.05	91.53	90.63%
5	Q296H	1.72	−9.28	90.07	90.79%
6	R92Q	1.70	−9.12	91.02	90.63%
7	R220K	1.70	−9.21	91.27	90.63%
8	Wild Type	1.70	−9.17	91.27	90.63%

**Table 2:** Physiochemical properties of the anticipated mutant models.

Sr No	Mutated Structures	Theoretical pI	GRAVY	AI
1	G465R	7.68	−0.304	82.32
2	L211F	7.31	−0.297	81.43
3	L478P	7.31	−0.307	81.48
4	P152S	7.31	−0.293	82.32
5	Q296H	7.31	−0.288	82.32
6	R92Q	6.98	−0.293	82.32
7	R220K	7.30	−0.294	82.32
8	Wild Type	7.31	−0.295	82.32

AI: Aliphatic index; GRAVY: grand average of hydropathicity.

**Table 3:** The top five predicted binding pocket scores and amino acid residues.

Rank	Name	Score	Probability	Amino Acid Residues
1	Pocket1	9.23	0.54	Ile_100, Ser_102, Ala_162, Cys_163, Gly_164, Ser_165, Phe_217, His_218, Gly_219, Arg_220, Glu_293, Asp_326, Val_328, Gln_329, Gln_330, Ser_356, Lys_357, Met_360
2	Pocket2	3.64	0.143	Glu_169, Leu_172, Lys_173, Phe_176, Met_177, Cys_205, Pro_206, Asp_207, Tyr_208, Gly_223, Phe_241, Trp_243
3	Pocket3	3.37	0.124	Val_463, His_72, Phe_73, Cys_75, Leu_85, Asp_95, Tyr_97, Gln_99
4	Pocket4	3.01	0.1	Met_177, Arg_180, Ser_181, Arg_184, Phe_189, Leu_194, Cys_197, Gly_204, Pro_206, Asn_373, Arg_377
5	Pocket5	2.96	0.097	Ser_102, Val_103, Pro_104, Gly_106, Ser_108, His_109, Leu_112, Ile_116, Met_360, Lys_388, Leu_391

**Table 4:** The PDBeFold and Gibbs free energy values of predicted models compared with the wild type.

Sr No	Mutated Models	Consensus RMSD	Gibbs Free Energy Change (ΔG)
1	Wild type	0.0010	–
2	R92Q	0.0026	1.3152
3	P152S	0.0016	1.6217
4	L211F	0.0024	1.3274
5	R220K	0.0013	0.2570
6	Q296H	0.0041	0.1922
7	G465R	0.0015	0.6539
8	L478P	0.0037	2.9431

## Data Availability

Not applicable.

## Supplementary Materials

The following supporting information can be downloaded at: https://www.mdpi.com/article/10.3390/ijms241310933/s1.

## References

[1] Hegde A.U., Karnavat P.K., Vyas R., DiBacco M.L., Grant P.E., Pearl P.L. (2019). GABA Transaminase Deficiency With Survival Into Adulthood. J. Child Neurol..

[2] Oshi A., Alfaifi A., Seidahmed M.Z., Al Hussein K., Miqdad A., Samadi A., Abdelbasit O.J. (2021). GABA transaminase deficiency. Case report and literature review. Clin. Case Rep..

[3] Hampe C.S., Mitoma H., Manto M.J.G. GABA and Glutamate: Their Transmitter Role in the CNS and Pancreatic Islets; 2018. https://www.intechopen.com/chapters/57103.

[4] Ghit A., Assal D., Al-Shami A.S., Hussein D.E. (2021). GABA(A) receptors: Structure, function, pharmacology, and related disorders. J. Genet. Eng. Biotechnol..

[5] Herbison A.E., Moenter S.M. (2011). Depolarising and hyperpolarising actions of GABA(A) receptor activation on gonadotrophin-releasing hormone neurones: Towards an emerging consensus. J. Neuroendocrinol..

[6] Sallard E., Letourneur D., Legendre P. (2021). Electrophysiology of ionotropic GABA receptors. Cell Mol. Life Sci..

[7] Patel A., Gokulgandhi M., Khurana V., Mitra A.K., Mitra A.K. (2013). 5-Transporters and receptors in the posterior segment of the eye. Ocular Transporters and Receptors.

[8] Magnaghi V. (2007). GABA and neuroactive steroid interactions in glia: New roles for old players?. Curr. Neuropharmacol..

[9] Kang S., Liu L., Wang T., Cannon M., Lin P., Fan T.W.M., Scott D.A., Wu H.-J.J., Lane A.N., Wang R. (2022). GAB functions as a bioenergetic and signalling gatekeeper to control T cell inflammation. Nat. Metab..

[10] Silverman R.B. (2018). Design and Mechanism of GABA Aminotransferase Inactivators. Treatments for Epilepsies and Addictions. Chem. Rev..

[11] Storici P., De Biase D., Bossa F., Bruno S., Mozzarelli A., Peneff C., Silverman R.B., Schirmer T. (2004). Structures of gamma-aminobutyric acid (GABA) aminotransferase, a pyridoxal 5’-phosphate, and [2Fe-2S] cluster-containing enzyme, complexed with gamma-ethynyl-GABA and with the antiepilepsy drug vigabatrin. J. Biol. Chem..

[12] Aldana B.I., Zhang Y., Jensen P., Chandrasekaran A., Christensen S.K., Nielsen T.T., Nielsen J.E., Hyttel P., Larsen M.R., Waagepetersen H.S. (2020). Glutamate-glutamine homeostasis is perturbed in neurons and astrocytes derived from patient iPSC models of frontotemporal dementia. Mol. Brain.

[13] Yogeswara I.B.A., Maneerat S., Haltrich D. (2020). Glutamate Decarboxylase from Lactic Acid Bacteria-A Key Enzyme in GABA Synthesis. Microorganisms.

[14] Silverman R.B. (2012). The 2011 EB Hershberg Award for important discoveries in medicinally active substances:(1 S, 3 S)-3-amino-4-difluoromethylenyl-1-cyclopentanoic acid (CPP-115), a GABA aminotransferase inactivator and new treatment for drug addiction and infantile spasms. J. Med. Chem..

[15] Huang D., Alexander P.B., Li Q.-J., Wang X.-F. (2023). GABAergic signaling beyond synapses: An emerging target for cancer therapy. Trends Cell Biol..

[16] Feng Y., Wei Z.-H., Liu C., Li G.-Y., Qiao X.-Z., Gan Y.-J., Zhang C.-C., Deng Y.-C. (2022). Genetic variations in GABA metabolism and epilepsy. Seizure.

[17] Lee H.H.C., McGinty G.E., Pearl P.L., Rotenberg A. (2022). Understanding the Molecular Mechanisms of Succinic Semialdehyde Dehydrogenase Deficiency (SSADHD): Towards the Development of SSADH-Targeted Medicine. Int. J. Mol. Sci..

[18] Koenig M.K., Hodgeman R., Riviello J.J., Chung W., Bain J., Chiriboga C.A., Ichikawa K., Osaka H., Tsuji M., Gibson K.M. (2017). Phenotype of GABA-transaminase deficiency. Neurology.

[19] Yasir M., Park J., Han E.-T., Park W.S., Han J.-H., Kwon Y.-S., Lee H.-J., Hassan M., Kloczkowski A., Chun W. (2023). Investigation of Flavonoid Scaffolds as DAX1 Inhibitors against Ewing Sarcoma through Pharmacoinformatic and Dynamic Simulation Studies. Int. J. Mol. Sci..

[20] Yasir M., Park J., Han E.-T., Park W.S., Han J.-H., Kwon Y.-S., Lee H.-J., Chun W. (2023). Computational Exploration of Licorice for Lead Compounds against Plasmodium vivax Duffy Binding Protein Utilizing Molecular Docking and Molecular Dynamic Simulation. Molecules.

[21] Willard L., Ranjan A., Zhang H., Monzavi H., Boyko R.F., Sykes B.D., Wishart D.S. (2003). VADAR: A web server for quantitative evaluation of protein structure quality. Nucleic Acids Res..

[22] Studio D.J.A. (2008). Discovery Studio. https://scholar.google.com/scholar?hl=en&as_sdt=0%2C5&q=Studio%2C+Discovery.+%22Discovery+studio.%22+Accelrys+%5B2.1%5D+%282008%29.&btnG=.

[23] Pettersen E.F., Goddard T.D., Huang C.C., Couch G.S., Greenblatt D.M., Meng E.C., Ferrin T.E. (2004). UCSF Chimera—A visualization system for exploratory research and analysis. J. Comput. Chem..

[24] Yasir M., Park J., Han E.T., Park W.S., Han J.H., Kwon Y.S., Lee H.J., Hassan M., Kloczkowski A., Chun W. (2023). Exploration of Flavonoids as Lead Compounds against Ewing Sarcoma through Molecular Docking, Pharmacogenomics Analysis, and Molecular Dynamics Simulations. Molecules.

[25] Jendele L., Krivak R., Skoda P., Novotny M., Hoksza D.J. (2019). PrankWeb: A web server for ligand binding site prediction and visualization. Nucleic Acids Res..

[26] Valdés-Tresanco M.S., Valdés-Tresanco M.E., Valiente P.A., Moreno E. (2021). gmx_MMPBSA: A New Tool to Perform End-State Free Energy Calculations with GROMACS. J. Chem. Theory Comput..

[27] Velankar S., Best C., Beuth B., Boutselakis C.H., Cobley N., Sousa Da Silva A.W., Dimitropoulos D., Golovin A., Hirshberg M., John M. (2010). PDBe: Protein Data Bank in Europe. Nucleic Acids Res..

[28] Witvliet D.K., Strokach A., Giraldo-Forero A.F., Teyra J., Colak R., Kim P.M. (2016). ELASPIC web-server: Proteome-wide structure-based prediction of mutation effects on protein stability and binding affinity. Bioinformatics.

