# Supplementary material for: Computational Exploration of the Effects of Mutations on GABA Aminotransferase in GABA Aminotransferase Deficiency
Source: Int J Mol Sci. 2023 Jun 30;24(13):10933. doi: 10.3390/ijms241310933 (PMC10342115; doi:10.3390/ijms241310933)
Supplement: Supplementary file 1 [file ijms-24-10933-s001.zip › ijms-2453176-supplementary.pdf]

International Journal of
Molecular Sciences

MDPI

Computational Exploration of the Effects of Mutations on GABA Aminotransferase in GABA Aminotransferase Deficiency

Muhammad Yasir [1], Jinyoung Park [1], Eun-Taek Han [2], Won Sun Park [3], Jin-Hee Han [2], Yong-Soo Kwon [4], Hee-Jae Lee [1] and Wanjoo Chun [1,*]

1 Department of Pharmacology, Kangwon National University School of Medicine, Chuncheon 24341, Republic of Korea; yasir.khokhar1999@gmail.com (M.Y.); jinyoung0326@kangwon.ac.kr (J.P.); heejaelee@kangwon.ac.kr (H.-J.L.)
2 Department of Medical Environmental Biology and Tropical Medicine, Kangwon National University School of Medicine, Chuncheon 24341, Republic of Korea; ethan@kangwon.ac.kr (E.-T.H.); han.han@kangwon.ac.kr (J.-H.H.)
3 Department of Physiology, Kangwon National University School of Medicine, Chuncheon 24341, Republic of Korea; parkws@kangwon.ac.kr
4 College of Pharmacy, Kangwon National University School of Medicine, Chuncheon 24341, Republic of Korea; yskwon@kangwon.ac.kr
* Correspondence: wchun@kangwon.ac.kr; Tel.: +82-33-250-8853

Table S1. Binding Pocket score values and the predicted amino acid residues by ParankWeb.

Sr no	Name	Score	Probability	Amino acid residues
1	pocket1	9.23	0.54	A_100 A_102 A_162 A_163 A_164 A_165 A_217 A_218 A_219 A_220 A_293 A_326 A_328 A_329 A_330 A_356 A_357 A_360
2	pocket2	3.64	0.143	A_169 A_172 A_173 A_176 A_177 A_205 A_206 A_207 A_208 A_223 A_241 A_243
3	pocket3	3.37	0.124	A_463 A_72 A_73 A_75 A_85 A_95 A_97 A_99
4	pocket4	3.01	0.1	A_177 A_180 A_181 A_184 A_189 A_194 A_197 A_204 A_206 A_373 A_377
5	pocket5	2.96	0.097	A_102 A_103 A_104 A_106 A_108 A_109 A_112 A_116 A_360 A_388 A_391
6	pocket6	2.93	0.095	A_298 A_450 A_451 A_454 A_458 A_463 A_464 A_465 A_466 A_72 A_97
7	pocket7	2.73	0.083	A_131 A_132 A_139 A_158 A_159 A_160 A_372 A_378 A_379 A_383
8	pocket8	2.5	0.07	A_251 A_255 A_294 A_295 A_303 A_304 A_305 A_306 A_340 A_435 A_436
9	pocket9	2.32	0.06	A_178 A_182 A_317 A_320 A_321 A_322 A_351 A_367 A_369
10	pocket10	1.84	0.034	A_214 A_215 A_248 A_249 A_250 A_299 A_300 A_302 A_432 A_468 A_469 A_471
11	pocket11	1.48	0.02	A_163 A_165 A_166 A_221

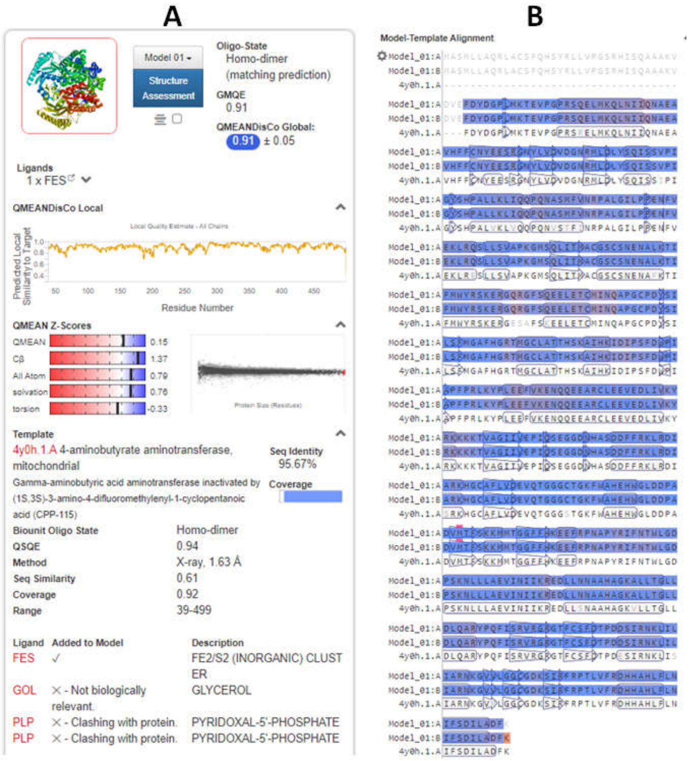

Figure S1. The predicted model of human GABA-AT with reference X-ray structure of *Sus Scrofa* with 1.63Å resolution is predicted in A while the sequence alignment of *Sus Scrofa* Human GABA-AT is depicted in B.

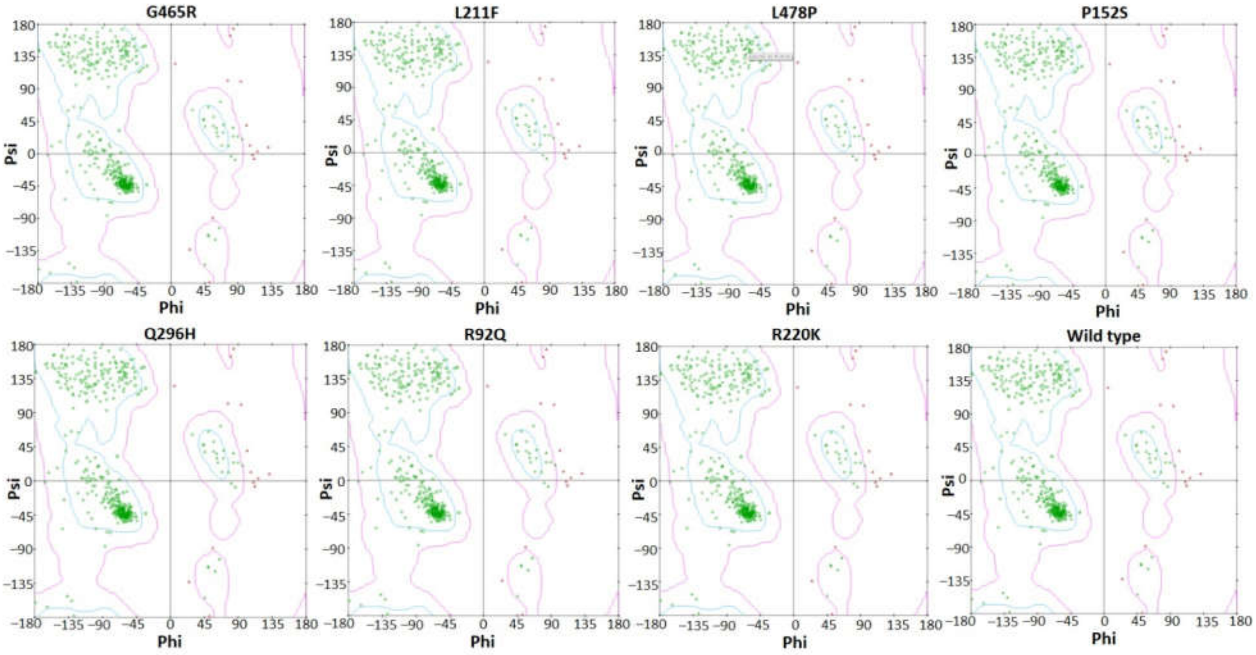

Figure S2. Ramachandran graphs of all mutated models in comparison with wild type. The amino acid residues are colored as green (green dots) while the favorable region boundary line is mentioned by blue. Additionally, and disallowed region is highlighted by pink color.

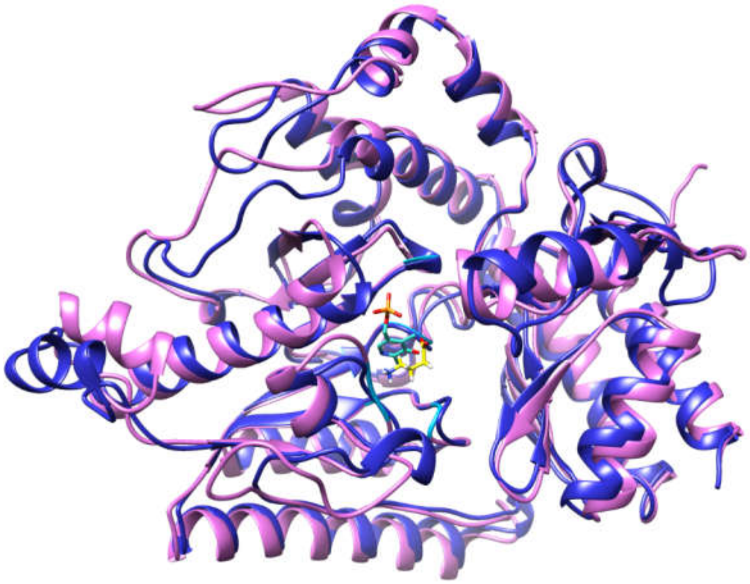

Figure S3. The superimposition of our predicted model of GABA-AT and the binding pocket with the already known structure of GABA-AT *Sus Scrofa* (PDBID-4Y0H). **Pyridoxal-5'-phosphate which was already bounded to** 4Y0H is colored as turquoise. Moreover, the GABA bound to predicted human GABA-AT model is colored yellow. Additionally, 4Y0H is colored as medium blue while the human GABA-AT is colored as an orchid.

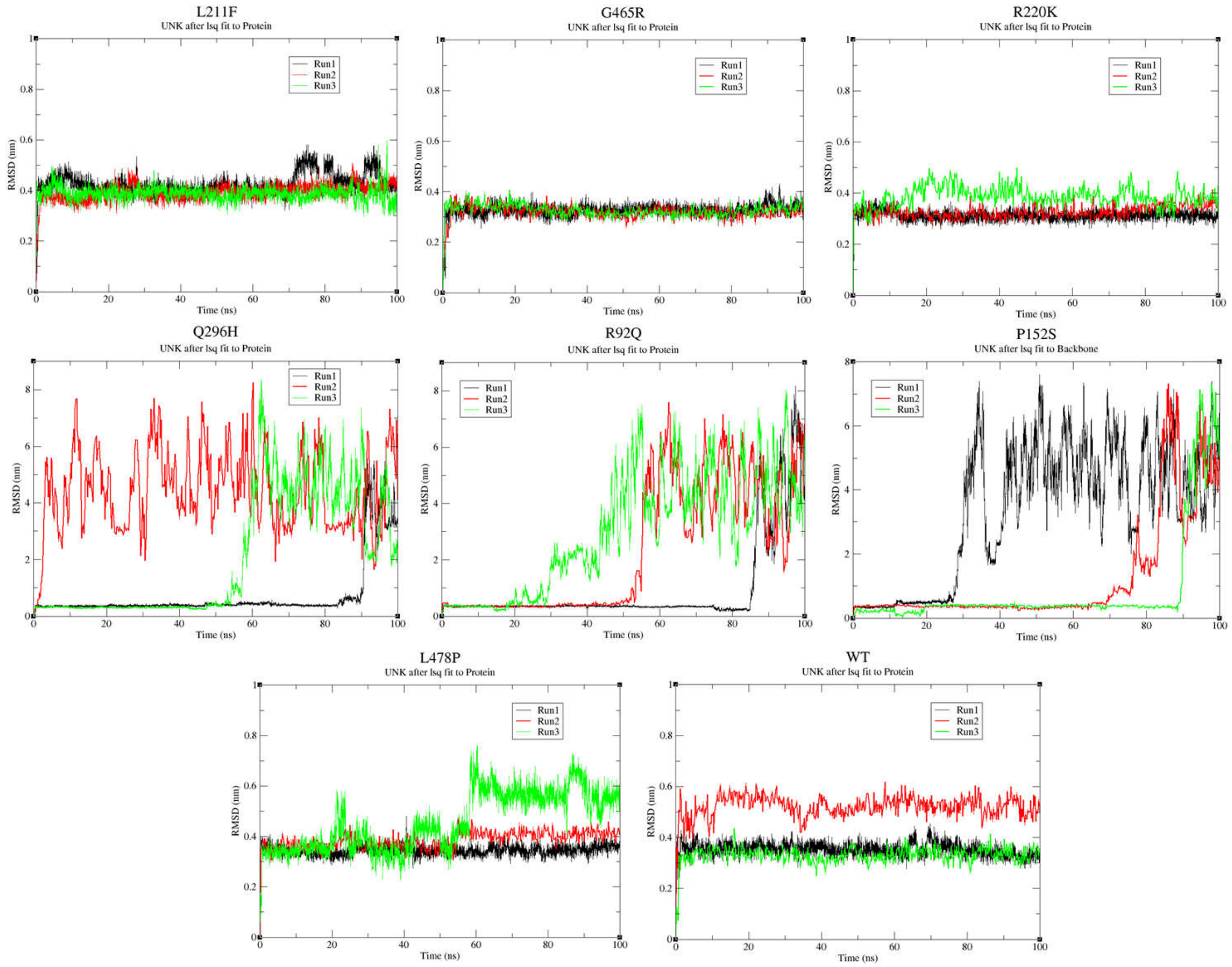

Figure S4. The graphical depiction of triplicates for seven mutated models in comparison with the wild type (WT). The run1, run2, and run3 are colored separately as black, red, and green respectively.
